# Central Inhibition of Tumor Necrosis Factor Alpha Reduces Hypertension by Attenuating Oxidative Stress in the Rostral Ventrolateral Medulla in Renovascular Hypertensive Rats

**DOI:** 10.3389/fphys.2019.00491

**Published:** 2019-04-30

**Authors:** Alynne Carvalho-Galvão, Drielle D. Guimarães, José L. De Brito Alves, Valdir A. Braga

**Affiliations:** ^1^Biotechnology Center, Federal University of Paraíba, João Pessoa, Brazil; ^2^Department of Physiology and Pharmacology, Karolinska Institute, Stockholm, Sweden; ^3^Department of Nutrition, Health Sciences Center, Federal University of Paraíba, João Pessoa, Brazil

**Keywords:** renovascular hypertension, TNF-α, sympathetic nervous system, superoxide, baroreflex, proinflammatory cytokines

## Abstract

Inflammation in the central nervous system is being considered a key player linked to neurogenic hypertension. Using combined *in vivo* and *in vitro* approaches, we investigated the effects of central inhibition of TNF-α on blood pressure, sympathetic tone, baroreflex sensitivity, and oxidative stress in the rostral ventrolateral medulla (RVLM) of rats with 2-kidney-1-clip (2K1C) renovascular hypertension. Continuous infusion of pentoxifylline, a TNF-α inhibitor, into the lateral ventricle of the brain for 14 consecutive days reduced blood pressure and improved baroreflex sensitivity in renovascular hypertensive rats. Furthermore, central TNF-α inhibition reduced sympathetic modulation and blunted the increased superoxide accumulation in the RVLM of 2K1C rats. Our findings suggest that TNF-α play an important role in the maintenance of sympathetic vasomotor tone and increased oxidative stress in the RVLM during renovascular hypertension.

## Introduction

Renovascular hypertension is characterized by the stenosis of one or two renal artery and is considered a secondary form of hypertension. Classical experiments performed by Goldblatt et al. (1934) demonstrated that the stenosis of one kidney induces massive renin release, leading to the activation of the renin-angiotensin-system. Studies by your group and others have documented that angiotensin-II (Ang-II) acting on the central nervous system is a key player in the development of the disease (Oliveira-Sales et al., 2010; Botelho-Ono et al., 2011; Burmeister et al., 2011).

It is well documented that inflammation in cerebral blood vessels induced by Ang-II plays a key role in the early stages of arterial hypertension (Winklewski et al., 2015). Circulating Ang-II activates leukocytes that produce proinflammatory cytokines such as IL-1 β, IL-6, and TNF-α (Shi et al., 2010; Waki et al., 2011). TNF-α plays an important role in the immune system and in the propagation of inflammation. Moreover, via its TNFR1 receptor, TNF-α can directly induce oxidative stress by the activation of reactive oxygen species (ROS)-producing enzymes, such as NADPH oxidase, contributing to neurodegeneration (Li et al., 2014). Thus, Ang-II-dependent activation of the sympathetic nervous system and subsequent hypertensive responses might be associated with high levels of cytokines and oxidative stress in the central nervous system (CNS).

It is well accepted that circulating levels of Ang-II modulate angiotensinergic projections from the subfornical organ (SFO), an important circumventricular organ, to neurons in the paraventricular nucleus of the hypothalamus (PVN), leading to increases in sympathoexcitatory inputs to the spinal cord and to pre-sympathetic neurons in the rostral ventrolateral medulla (RVLM) (Zimmerman et al., 2004; Braga et al., 2011; Burmeister et al., 2011). Chronic inhibition of the angiotensin-converting enzyme in the paraventricular nucleus of the hypothalamus (PVN) attenuates both sympathoexcitation and ROS accumulation, and modulates expression of cytokines (decreasing TNF-α, IL-1β, and IL-6) in the RVLM during renovascular hypertension (Li et al., 2014). Those effects might be related to direct neuronal projections from the PVN to the RVLM (Braga et al., 2011). However, direct central inhibition of pro-inflammatory mediators, such as TNF-α, on sympathetic activity, baroreflex sensitivity and ROS accumulation in the RVLM of renovascular hypertensive rats has not been investigated. Therefore, considering that the 2-kidney-1-clip experimental model of hypertension holds a neurogenic component and central oxidative stress associated to the maintenance of hypertension, we tested the hypothesis that central inhibition of TNF-α attenuates oxidative stress within the RVLM and reduces sympathetic modulation and blood pressure in renovascular hypertensive rats.

## Materials and Methods

### Animals

Male Wistar rats (150–300 g) were housed in a temperature-controlled room, exposed to a 12:12 h light–dark cycle with free access to standard rat chow (Labina^®^, Purina, Paulínia, Brazil) and water. Experimental procedures were performed in accordance to National Institutes of Health guidelines and protocols were approved by Federal University of Paraíba Animal Care and Use Committee (CEUA/UFPB n. 101/15). Animals were divided in three groups: Sham-operated rats (Sham, *n* = 8), 2-kidney-1-clip (2K1C, *n* = 8), and 2K1C + pentoxifylline (2K1C + PTX, *n* = 6). Pentoxifylline (Sigma-Aldrich, St. Louis, MO, United States), a TNF-α inhibitor, was given during 14 consecutive days (30 nmol/μL/h by intracerebroventricular infusion, ICV). Pentoxifylline dose was chosen based on previous studies (Wu et al., 2012).

### General Experimental Protocol

**FIGURE 1:**
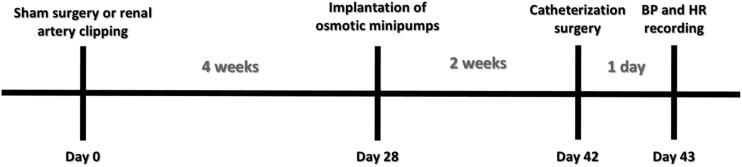
Experimental design.

All the procedures described in the following sections were performed in all animals from each group at the end of the sixth week after 2K1C or sham surgery. In the end of the fourth week after induction of renovascular hypertension, animals from the 2K1C + PTX group underwent implantation of osmotic minipumps (Alzet 2002; Durect Co., Cupertino, CA, United States) with pentoxifylline (30 nmol/μL/h) into the lateral ventricle, which remained for 14 days until the end of the sixth week (Figure 1). In order to reduce potential peripheral effects of pentoxifylline, the dose of pentoxifylline chosen was equivalent to 6.0–7.0 × 10^–6^ mg/kg every 24 h. A hypertensive control group (2K1C) underwent the same surgical procedure without osmotic minipump implantation.

### Renal Artery Clipping Procedure

Renovascular hypertension (2K1C model) was induced in rats as previously described (Cavalcanti et al., 2016). Under combined ketamine (Cetamin, Syntec, Cotia, Brazil) and xylazine (Anasedan, Cevo, Paulínia, Brazil) anesthesia (75 and 10 mg/kg, intraperitoneal, IP, respectively), a midline abdominal incision was made. The right renal artery was exposed and isolated over a short segment by blunt dissection. A U-shaped silver clip (0.2 mm internal gap) was placed over the vessel at a site proximal to the abdominal aorta and the wound closed and sutured. A sham procedure, which entailed the entire surgery except for renal artery clipping, served as control.

### Intracerebroventricular Infusion Using Osmotic Minipumps

After 4 weeks of renal artery clipping, animals from the 2K1C + PTX group received implantation of an osmotic minipump (model 2002, ALZET, pumping rate ± 0.1 μl/h) with a brain infusion kit (brain infusion kit 2, ALZET) for ICV infusion of pentoxifylline (30 nmol/μL/h). To minimize the chance of occlusion and to allow stabilization of the delivery system, the pump was placed in sterile 0.9% saline at 37°C overnight before implantation as recommended by the manufacturer. Procedures for implantation of the pump were adopted from previously published protocol (Carvalho-Galvao et al., 2018b). Briefly, animals were anesthetized with a combination of ketamine and xylazine (75 and 10 mg/kg, IP, respectively), and placed on the stereotaxic apparatus. A midline incision was made to expose dorsal surface of the skull. A burr hole was created at 0.9 mm posterior to the bregma and 1.5 mm lateral to the midline, and a cannula was inserted to the right lateral ventricle 4.0 mm below the pial surface. The cannula was sealed and connected to an osmotic minipump by polyethylene catheter. The pump was placed subcutaneously in the dorsal region of the back. In 2K1C and sham groups, a sham procedure was performed and only the burr hole was created.

### Blood Pressure and Heart Rate Recordings

Six weeks after the induction of hypertension (or sham surgery), rats were anesthetized with a combination of ketamine and xylazine (75 and 10 mg/kg, IP, respectively) for catheters implantation and direct hemodynamic measurements. Polyethylene catheters were inserted into the abdominal aorta and inferior vena cava through femoral artery and vein for arterial pressure recordings and drug injections, respectively. Blood pressure and heart rate measurements were taken 24 h after catheter implantation in conscious rats using a pressure transducer coupled to an acquisition system (PowerLab; ADInstruments, Castle Hill, NSW, Australia) connected to a computer running LabChart 7.0 software (ADInstruments, Castle Hill, NSW, Australia).

### Baroreflex Sensitivity Test

After 50 min of blood pressure and heart rate baseline recordings, baroreflex was activated using classical vasoactive drugs based on the modified Oxford Method according to Braga et al. (2008). Phenylephrine (8 μg/Kg) and sodium nitroprusside (25 μg/Kg) were given as intravenous bolus injection in animals from each group. A 15-min interval was allowed between phenylephrine and sodium nitroprusside injections. Reflex changes in heart rate produced by vasoactive drugs administration were quantified and plotted as changes in heart rate over changes in mean arterial pressure (ΔHR/ΔMAP). Data were analyzed by linear regression using Prism 6 (GraphPad Software, Inc., San Diego, CA, United States) and the slope of linear regression provided baroreflex gain for each animal. In this study, spontaneous baroreflex sensitivity (SBRS) was also calculated through the sequence method by the computer software CardioSeries (v2.4). A baroreflex sequence was defined as a sequence of at least four heart beats in which both systolic arterial pressure and pulse interval increased (up sequences) or decreased (down sequences) as previously described (Braga et al., 2008).

### Evaluation of Sympathetic Tonus to the Vasculature

In a separate group of rats, the sympathetic vascular tone was evaluated by an intravenous injection of hexamethonium (30 mg/Kg, Sigma-Aldrich, São Paulo, Brazil), a ganglionic blocker. The magnitude of the fall in blood pressure yield the contribution of the sympathetic tone to sustain blood pressure at baseline level.

### Power Spectral Analysis of Systolic Arterial Pressure

Systolic arterial pressure (SAP) signals were evaluated by power spectral analysis. In our study, we were particularly interested in the low-frequency component of SAP signals (LF, 0.25–0.8 Hz), that is representative of the modulatory effects of sympathetic tone and was reported to take origin from the RVLM (Kuo et al., 1997). To reach this goal, beat-by-beat time series of SAP were extracted from baseline cardiovascular recordings (10 min epochs) of pulsatile arterial pressure of rats in each group and the variability of these series was assessed using Fast Fourier Transform spectral analysis (Cardioseries Software v2.4) (Tezini et al., 2013).

### Measurement of Superoxide Anion

Considering that the accumulation of superoxide anions induced by Ang-II in RVLM is critical for the pathogenesis of hypertension, leading to an increase in sympathetic activity (Braga et al., 2011), we measured the levels of superoxide anions in that region. At the end of the *in vivo* protocols, under ketamine and xylazine anesthesia, animals underwent decapitation, brains were removed, quickly frozen, embedded in optimum cutting temperature compound (Tissue-Tek, Sakura, Japan), cryosectioned (30 μm, coronal section) and mounted directly onto chilled microscope slides. Sections were thawed at room temperature, rehydrated with 1× phosphate buffered saline (PBS), and incubated for 5 min in the dark with the superoxide specific fluorogenic probe dihydroethidium (DHE; 1 μM, Dihydroethidium, Sigma-Aldrich, São Paulo, Brazil). After washing with 1× PBS, DHE fluorescence was visualized as previously described (Braga, 2010b; Alves et al., 2015). RGB fluorescence images were loaded into ImageJ software and converted to 8-bit gray-scale before subtracting background fluorescence equivalently for all images (setting the threshold to 50% maximum intensity). The mean fluorescence was quantified and expressed relative to values obtained for control rats (sham group).

### Determination of Kidneys and Heart Weight

Heart and kidneys were collected and weighted. Total organ mass (mg) were normalized by the body weight (g) giving an organ weight/body weight ratio index (ow/bw). The index was adopted to prevent variations among different animal sizes.

### Statistical Analysis

Results are expressed as mean ± SEM. Data were analyzed by one-way ANOVA followed by Tukey’s *post hoc* when appropriate. Statistical analyses were performed using Prism 6 (GraphPad, La Jolla, CA, United States) and the differences were considered significant when *P* < 0.05.

## Results

### Body and Organs Weight

As shown in Table 1, right clipped kidneys from both 2K1C groups presented a reduction in the kidney mass index when compared to right kidneys from sham-operated rats. In addition, the left non-clipped kidney mass index was increased in both 2K1C groups when compared to left kidney from sham-operated rats. It is important to highlight that the success rate of the 2K1C procedure under our experimental conditions exceeded 95%. Absolute values for body and organ weights were also provided in Table 1.

**TABLE 1:** Body and organs weight values.

	Initial body weight (g)	Final body weight (g)	Right kidney/final body mass (mg/g)	Left kidney/final body mass (mg/g)	Heart/final body mass (mg/g)	Right kidney (g)	Left kidney (g)	Heart (g)
Sham	169.1 ± 6.2	239.3 ± 5.1*	4.3 ± 0.14*	4.2 ± 0.13*	3.6 ± 0.24	1.0 ± 0.03	1.0 ± 0.04*	0.86 ± 0.05*
2K1C	181.2 ± 5.2	272.5 ± 13.7	3.37 ± 0.22^#^	4.8 ± 0.17	4.3 ± 0.39	0.91 ± 0.07^#^	1.3 ± 0.05	1.1 ± 0.07
2K1C + PTX	176 ± 5.9	284.7 ± 7.9	3.32 ± 0.17^#^	4.8 ± 0.06	3.9 ± 0.22	0.92 ± 0.02^#^	1.4 ± 0.02	1.1 ± 0.04

*P < 0.05 vs. other groups. ^#^*P* < 0.05 vs. compared to the left organ from the same group.

### Central Inhibition of TNF-α Reduces Blood Pressure in Renovascular Hypertensive Rats

**FIGURE 2:**
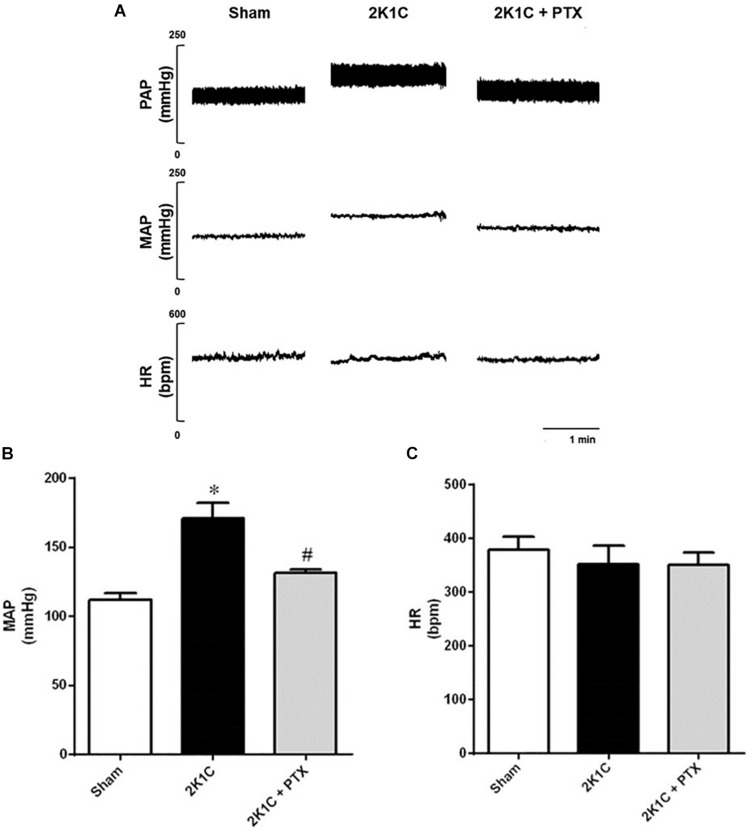
Central inhibition of TNF-α reduces blood pressure in renovascular hypertensive rats. **(A)** Original tracings from one representative animal of each group (Sham, 2K1C, 2K1C + PTX) showing pulsatile arterial pressure (PAP), mean arterial pressure (MAP), and heart rate (HR). **(B)** Effects of central inhibition of TNF-α with pentoxifylline (PTX) for 14 days in 2K1C rats on MAP (*P = 0.0001 vs. sham; ^#^*P* = 0.0175 vs. 2K1C). **(C)** Effects of central inhibition of TNF-α with PTX during 14 days in 2K1C rats on HR.

Mean arterial pressure (MAP) and heart rate (HR) data are summarized in Figure 2. Figure 2A shows original tracings of pulsatile arterial pressure (PAP), MAP, and HR from one representative animal of each group. As expected, rats from 2K1C group presented a significant increase in mean blood pressure after 6 weeks of renal artery clipping compared to sham-operated rats (171 ± 11 mmHg vs. 113 ± 5 mmHg, *P* = 0.0001, *n* = 8; Figure 2B). The central inhibition of TNF-α with pentoxifylline (30 nmol/μL/h, ICV) for 14 days in rats with renovascular hypertension (*n* = 6) reduced MAP when compared to non-treated hypertensive rats (*n* = 8) (131 ± 2 mmHg vs. 171 ± 11 mmHg; *P* = 0.0175; Figure 2B). Of note, HR was not different among groups as shown in Figure 2C.

### Baroreflex Sensitivity Was Restored in Renovascular Hypertensive Rats After Central Inhibition of TNF-α

**FIGURE 3:**
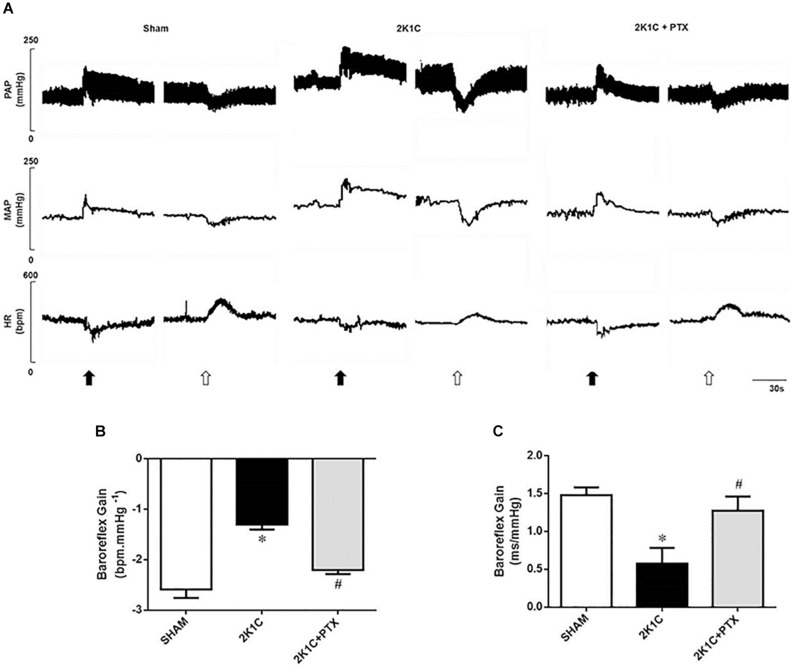
Central inhibition of TNF-α restores baroreflex sensitivity in renovascular hypertensive rats. **(A)** Original tracings from one representative animal of each group (Sham, 2K1C, 2K1C + PTX) showing changes in pulsatile arterial pressure (PAP), mean arterial pressure (MAP), and heart rate (HR) in response to phenylephrine (8 μg/Kg, IV, black arrows) and sodium nitroprusside (25 μg/Kg, IV, open arrows). **(B)** Effects of central inhibition of TNF-α with pentoxifylline (PTX) for 14 days on drug-induced baroreflex sensitivity (baroreflex gain; *P = 0.0001 vs. sham; ^#^*P* = 0.0006 vs. 2K1C). **(C)** Effects of central inhibition of TNF-α with PTX for 14 days on spontaneous baroreflex sensitivity (baroreflex gain; *P = 0.0001 vs. sham; ^#^*P* = 0.0001 vs. 2K1C).

Original tracings from one representative animal from each group showing the changes in blood pressure and heart rate in response to the administration of vasoactive drugs are illustrated in Figure 3A. Animals from 2K1C group presented a reduction in baroreflex sensitivity when compared to sham group (−1.30 ± 0.10 vs. −2.59 ± 0.17 bpm mmHg^–1^, *P* = 0.0001, *n* = 8) as shown in Figure 3B. In 2K1C rats, central inhibition of TNF-α with pentoxifylline during 14 days (*n* = 6) restored the depressed baroreflex sensitivity to values that resembles those presented by sham-operated rats when compared to non-treated hypertensive rats (*n* = 8) (−2.20 ± 0.08 vs. −1.30 ± 0.10 bpm mmHg^–1^; *P* = 0.0006; Figure 3B). Spontaneous baroreflex sensitivity presented similar results (Figure 3C). Renovascular hypertensive rats presented reduced SBRS when compared to sham-operated rats (0.58 ± 0.08 vs. 1.48 ± 0.04 ms mmHg^–1^, *P* = 0.0001, *n* = 8). The central inhibition of TNF-α with pentoxifylline improves SBRS in 2K1C rats (*n* = 6) when compared to non-treated 2K1C rats (*n* = 8) (1.27 ± 0.15 vs. 0.58 ± 0.08 ms mmHg^–1^, *P* = 0.0001). These results show that central inhibition of TNF-α with pentoxifylline restored both spontaneous and drug-induced baroreflex sensitivity of 2K1C rats.

### Central Inhibition of TNF-α Reduces Sympathetic Tone in Renovascular Hypertensive Rats

**FIGURE 4:**
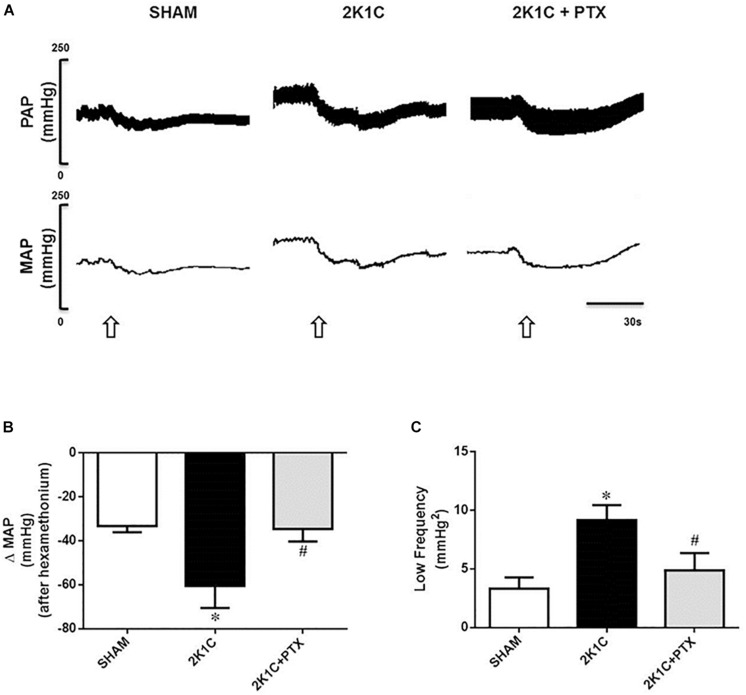
Central inhibition of TNF-α reduces sympathetic tone in renovascular hypertensive rats. **(A)** Original tracings from one representative animal of each group (Sham, 2K1C, 2K1C + PTX) showing changes in pulsatile arterial pressure (PAP), and mean arterial pressure (MAP) in response to ganglionic blockade with hexamethonium (30 μg/Kg, i.v., open arrows). **(B)** Effects of central inhibition of TNF-α with pentoxifylline (PTX) for 14 days on evaluation of delta change of the MAP after blockade with hexamethonium (*P = 0.0002 vs. sham; ^#^*P* = 0.0093 vs. 2K1C). **(C)** Changes in the low-frequency component (LF) of the spectral analysis of systolic arterial pressure (SAP) from each group (*P = 0.0001 vs. sham; ^#^*P* = 0.0118 vs. 2K1C).

Original tracings from one representative animal of each group show changes in PAP and MAP after ganglionic blocker with hexamethonium (Figure 4A). The fall in MAP was larger in 2K1C group when compared to sham-operated group (−60 ± 5 vs. −33 ± 2 ΔmmHg, *P* = 0.0002, *n* = 8; Figure 4B). Central inhibition of TNF-α in 2K1C rats (*n* = 6) blunted the fall in blood pressure elicited by hexamethonium when compared to non-treated 2K1C rats (*n* = 8) (−34 ± 3 vs. −60 ± 5 ΔmmHg; *P* = 0.0093; Figure 4B). Furthermore, as observed in Figure 4C, 2K1C rats exhibited an increase in the LF component of the spectral analysis of SAP when compared to sham-operated rats (9.16 ± 0.52 vs. 3.32 ± 0.38 mmHg^2^, *P* = 0.0001, *n* = 8). This increase was reduced in 2K1C rats that received ICV infusion of pentoxifylline for 14 days (*n* = 6) when compared to non-treated 2K1C rats (*n* = 8) (4.90 ± 0.66 vs. 9.16 ± 0.52 mmHg^2^; *P* = 0.0118; Figure 4C). These data suggest the involvement of TNF-α in the control and maintenance of sympathetic tone in 2K1C model.

### Superoxide Accumulation in the RVLM Seems to Involve TNF-α in Renovascular Hypertensive Rats

**FIGURE 5:**
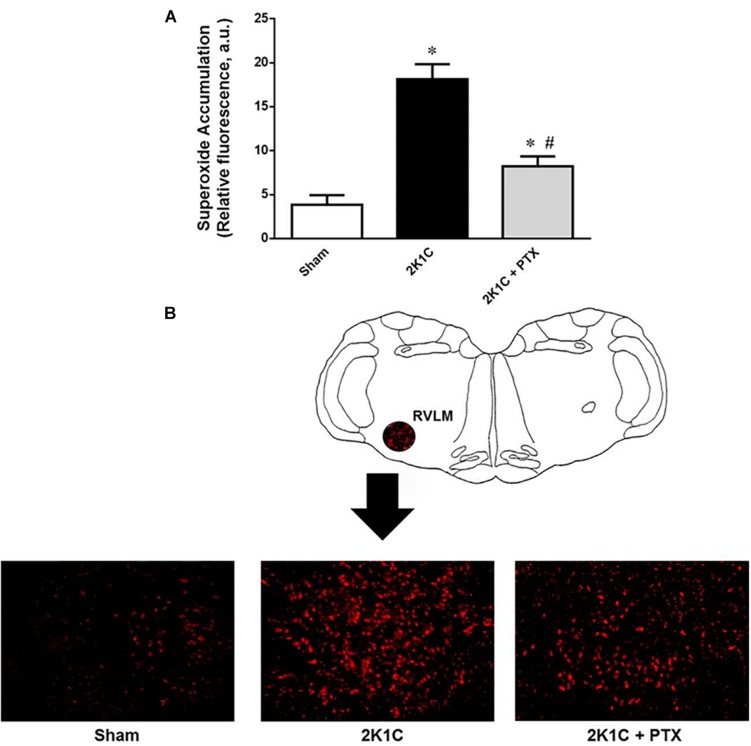
Central inhibition of TNF-α reduces superoxide accumulation in RVLM of renovascular hypertensive rats. **(A)** Superoxide accumulation measured by the dihydroethidium technique expressed as relative fluorescence (AU, arbitrary units) (*P = 0.0001 vs. sham; ^#^*P* = 0.0464 vs. 2K1C). **(B)** Schematic drawing showing the RVLM region and one representative image from each group illustrating the fluorescence that were quantified as an indication of the superoxide accumulation in the RVLM region.

The magnitude of superoxide accumulation in the RVLM was larger in 2K1C rats when compared to sham-operated rats (18 ± 2 vs. 4 ± 1 a.u., *P* = 0.0001, *n* = 8; Figure 5A). Central inhibition of TNF-α in renovascular hypertensive rats (*n* = 6) reduced superoxide formation in the RVLM when compared to non-treated 2K1C rats (*n* = 8) (8 ± 1 vs. 18 ± 2 a.u., *P* = 0.0464; Figure 4A). Representative images are shown in Figure 5B. These data suggest that TNF-α plays a role in the accumulation of superoxide in the RVLM during angiotensin-II-dependent renovascular hypertension.

## Discussion

Our findings provide evidence that the central inhibition of TNF-α reduces arterial pressure in renovascular hypertensive rats. We suggest that the attenuation of hypertension was associated with the reduction in sympathetic tone, improvement of baroreflex sensitivity and reduction in superoxide accumulation in the RVLM.

The experimental model of renovascular hypertension proposed by Goldblatt et al. (1934) mimics human renovascular hypertension. In that model, known as 2-kidney-1-clip model, the unilateral renal artery stenosis reduces renal perfusion and chronically stimulates the renin-angiotensin-aldosterone system (RAAS). The sympatho-excitatory responses triggered by RAAS activation are mainly caused by the Ang-II through AT1 receptors in both periphery and central nervous system (CNS) (Braga et al., 2011). In the CNS, Ang-II effects involve the activation of AT1 receptors in the subfornical organ (SFO), with recruitment of the enzyme NADPH oxidase and ROS formation, especially superoxide anion (O_2_^•–^), in the SFO, PVN, and RVLM (Griendling et al., 1994; Zimmerman et al., 2004; Braga et al., 2011; Shinohara et al., 2015). In addition to the already mentioned effects, Ang-II may also act as growth factor and as a stimulating agent of proinflammatory cytokines such as IL-6 and TNF-α (Kalra et al., 2002).

As inflammation has become an important component in the complex pathogenesis of arterial hypertension and cardiovascular diseases, the interaction between Ang-II and TNF-α within the brain might play an important role in the genesis and maintenance of hypertension (Kalra et al., 2002). In agreement with that hypothesis, our findings demonstrated that central inhibition of TNF-α by 14 consecutive days of pentoxifylline treatment, starting at 4 weeks after implantation of a silver clip in the renal artery, promoted a reduction in MAP compared to non-treated hypertensive rats, suggesting that TNF-α is acting on cardiovascular control areas in the central nervous system during the development of hypertension.

Although the arterial baroreflex has a major influence on the sympathetic nerve activity, during Ang-II-dependent hypertension, baroreflex sensitivity is reduced in order to allow sympathetic activity to increase independently of the concomitant rise in blood pressure (Brooks et al., 1993). Here we demonstrated that inhibition of TNF-α improved baroreflex sensitivity, probably contributing to the reduction in sympathetic activity and fall in blood pressure. Studies have shown the involvement of Ang-II on TNF-α activation and production of ROS within the brain (Sriramula et al., 2008). Of note, the stimulation of brain regions such as the PVN by TNF-α causes increases in sympathetic activity and raises in blood pressure (Shi et al., 2011). The mechanisms by which TNF-α is acting directly or indirectly to modulate the pre-sympathetic baro-sensitivity RVLM neurons in order to alter sympathetic tone is still matter for further investigation.

It is established that the sympathetic tone is increased in 2K1C hypertension model between the fourth and sixth weeks after silver clip implantation (Faber and Brody, 1983; Guild et al., 2012). Under our experimental conditions, ganglionic blockade with hexamethonium promoted a larger reduction in blood pressure of 2K1C rats, reflecting an increased sympathetic tone in animals with renovascular hypertension after 6 weeks. Interestingly, in 2K1C rats treated with pentoxifylline (i.c.v.) for 14 days, the fall in blood pressure elicited by hexamethonium was significantly reduced when compared to non-treated 2K1C rats. These data suggest that central TNF-α inhibition decreases sympathetic tone during the development and maintenance of renovascular hypertension. In addition, central inhibition of TNF-α reduced the increased power of the LF band of SAP spectrum in animals with renovascular hypertension, corroborating the ganglionic blocker studies. An important limitation of the study is that we evaluate the sympathetic function using classic but indirect approaches rather than recording sympathetic activity directly. One potential limitation of our study is that blood pressure was recorded 24 h after arterial and venous catheter implantation surgery. However, different of telemetry implantation surgery in the abdominal aorta by laparotomy, which is a major procedure that requires 5–7 days for a full recovery (Greene et al., 2007), implanting of blood vessels catheters via the femoral artery and vein could be considered an ambulatory procedure with just a minor incision on the inguinal region with no major cavity exposure. We have been very successful with this procedure over the last 15 years (Braga and Prabhakar, 2009; Braga, 2010a; Ribeiro et al., 2010; Botelho-Ono et al., 2011; Giusti et al., 2011; Franca-Silva et al., 2012; Guimaraes et al., 2012; Queiroz et al., 2012; Mendes-Junior et al., 2013; Alves et al., 2015; de Queiroz et al., 2015; Cavalcanti et al., 2016; Carvalho-Galvao et al., 2018a)

We have been investigating the mechanisms by which reductions in ROS lead to improvement of baroreflex sensitivity (Botelho-Ono et al., 2011; Giusti et al., 2011; Guimaraes et al., 2012; Monteiro et al., 2012; Queiroz et al., 2012; Mendes-Junior et al., 2013; de Queiroz et al., 2015). Although it is well documented that reducing ROS is important to restore baroreflex sensitivity during hypertension, our study is the first to show that the central inhibition of TNF-α with pentoxifylline promotes an improvement in baroreflex sensitivity in renovascular hypertensive rats, restoring the baroreflex sensitivity to values found in normotensive animals. Of note, our data show that pentoxifylline was able to improve both spontaneous and drug-induced baroreflex evaluations. Combining both analyses allows for the evaluation of the baroreflex function in a wide range of the reflex. This is in agreement with what we have recently shown (Guimaraes et al., 2019). We believe that this improvement in baroreflex sensitivity may be related to reduction in superoxide accumulation in RVLM promoted by central inhibition of TNF-α. Our findings are in agreement with Winklewski et al. (Winklewski et al., 2015) where authors documented that chronic inhibition of angiotensin-converting-enzyme (ACE) in the PVN of animals with renovascular hypertension triggered a reduction of sympathetic activity and ROS production, and modulated the expression of several cytokines in RVLM, such as TNF-α, showing the relation between the renin-angiotensin system and elevated levels of pro-inflammatory cytokines and oxidative stress in central regions.

The RVLM is a key brain region for the generation of sympathetic vasomotor activity. Therefore, we investigate the effects of central inhibition of TNF-α on the accumulation of superoxide in the RVLM. Our results showed an increase in superoxide production in the RVLM of non-treated hypertensive rats. It is known that the increase in superoxide in the RVLM promotes changes in ion channels, particularly Ca^+2^ and K^+^, changing the neuronal firing pattern in the RVLM, resulting in increased sympathetic activity and increased blood pressure (Hirooka, 2008). We demonstrated that central inhibition of TNF-α with pentoxifylline for 14 days reduces superoxide accumulation in RVLM of 2K1C hypertensive rats. Although the specific mechanism is still unknown, these results demonstrate the importance of TNF-α in the accumulation of superoxide in an Ang-II-dependent hypertension model, where this peptide is responsible for the activation of the NADPH oxidase, the main source of O_2_^•–^ in RVLM (Chan et al., 2007). Our findings extend the knowledge of a previous study reporting an attenuation of superoxide production mediated by Ang-II in mice treated with etanercept, a TNF-α blocker (Guzik et al., 2007). Although the underlying mechanisms related to the central inhibition of TNF-α remains unclear, our findings reinforce the putative link between sympathetic activity and proinflammatory cytokines, particularly TNF-α, in brain areas involved in cardiovascular control such as RVLM.

In addition to the brain mechanisms that clearly involves inflammation and ROS accumulation, specially in the RVLM as documented here, we cannot rule out the participation of the renal afferents contributing to the effects induced by augmented Ang-II in the 2K1C model. Actually, recent studies by Nishi et al. (2018) and by our group (Carlstrom and Braga, 2019) have documented and discussed, respectively, the important role of the renal afferent in the 2K1C model of hypertension. Authors demonstrated that renal denervation of the ischemic kidney blunts the increase of ROS in brain areas involved in cardiovascular control such as the PVN and the RVLM.

In conclusion, central inhibition of TNF-α reduces arterial pressure in renovascular hypertensive rats. The reduction in blood pressure seems to involve a combination of reduction in sympathetic tone, improvement of baroreflex sensitivity and reduction in superoxide accumulation in RVLM. For future studies, it will be important to evaluate the chronic inhibition of the TNF-α and its implications, including side effects, in the context of hypertension. While brain infusion is still far from the clinical use, it is imperative to seek for refining drug delivery in specific brain regions involved in the autonomic control of blood pressure in patients dealing with hypertension and other central nervous system-related diseases.

## Ethics Statement

Experimental procedures were performed in accordance with National Institutes of Health guidelines and protocols were approved by the Federal University of Paraíba Animal Care and Use Committee (CEUA/UFPB n. 101/15).

## Author Contributions

AC-G and VB figured and planned the experiments. AC-G and DG implemented the experiments. AC-G, JDBA, and VB analyzed the data. AC-G, DG, JDBA, and VB wrote the manuscript.

## Conflict of Interest Statement

The authors declare that the research was conducted in the absence of any commercial or financial relationships that could be construed as a potential conflict of interest.
